# Comparative Analysis of Roots from *Vicatia thibetica* de Boiss and *Angelica sinensis* Based on Chemical Composition, Antioxidant, Nitrite-Scavenging and Enzyme Inhibition Activities

**DOI:** 10.3390/molecules28041942

**Published:** 2023-02-17

**Authors:** Wenwen Tang, Yuan Chen, Fengxia Guo

**Affiliations:** 1 State Key Laboratory of Aridland Crop Science, College of Agronomy, Gansu Agricultural University, Lanzhou 730070, China; 2 Tongren Polytechnic College, Tongren 554300, China; 3 College of Life Science and Technology, Gansu Agricultural University, Lanzhou 730070, China

**Keywords:** Radix *Vicatia thibetica* de Boiss, chemical composition, tyrosinase inhibitory, thrombin and FXa inhibitory, antioxidant, nitrite-scavenging activity

## Abstract

Radix *Vicatia thibetica* de Boiss (RVT) is locally known as “Xigui” or “Dujiao-danggui” in Tibetan medicine and is often used as a substitute for Radix *Angelica sinensis* (RAS) in daily nourishing diets and clinical applications. In this study, we determined and compared the contents of polysaccharides, total coumarins, ferulic acid, total phenols, total flavonoids, chlorogenic acid, protein, and amino acids, and the composition of volatile oil in RVT and RAS. Biological activities, including antioxidants, scavenging of nitrite, inhibition of tyrosinase, thrombin, and coagulation FXa, were comparatively evaluated. Results showed that RVT contains more polysaccharides, phenols, flavonoids, proteins, glutamic acid, and lysine as compared to RAS. Among volatile compounds, 14 species are similar, and 20 species are different in RVT and RAS. Overall, among volatile compounds, the content of 3-N-Butylphthalide was higher, whereas the content of ligustilide was lower in RVT volatile oil. A significant difference was reported in the bioactivity of RVT and RAS. The biological activity of RVT had higher antioxidant, nitrite scavenging, and tyrosinase inhibitory activities, whereas it showed much lower thrombin and FXa inhibitory activities. Correlation analysis showed that the antioxidant, nitrite scavenging, and tyrosinase inhibitory activities were related to the phenol and flavonoid content, whereas the thrombin and FXa inhibitory activities were related to ferulic acid and volatile oil content. This study presents a comparative analysis of RAS and RVT’s chemical compositions of antioxidant, nitrite-scavenging, inhibition of tyrosinase, thrombin, and coagulation FXa activities. It was found that both RVT and RAS have their unique advantages, and RVT has the potential to be utilized as functional foods, cosmetics, and medical products.

## 1. Introduction

*Vicatia thibetica* de Boiss is a cone-shaped perennial herb of the Umbelliferae family. It is distributed in China, India, Pakistan, and Nepal, and locally used in China, which is the most distinctive medicinal and edible plant in Tibetan medicine. In China, it is abundantly present in Tibet, Sichuan, Yunnan, and other places [1]. Radix *Vicatia thibetica* de Boiss (RVT) is often used as a tonic in Tibetan medicine and has the functions of enhancing physical strength, improving digestion, nourishing blood, prolonging life, and improving malnutrition [2], which is one of the five root medicines of Tibetan medicine (Huangjing, Asparagus, Tibetan concave celery, Himalayan mirabilis, and Tribulus terrestris). In addition, it is often used as a nutritious vegetable to stew meat, chicken, and soup, and fresh products can also be eaten raw, having a sweet taste [1,2]. 

Radix *Angelica sinensis* (RAS) is the dried root of *Angelica sinensis* (Oliv.) Diels belong to the Umbelliferae family. *Angelica sinensis* (Oliv.) Diels is the official source of Danggui (in Chinese) in traditional Chinese medicine [3]. Materia Medica has the functions of nourishing blood, regulating menstruation and relieving pain, moisturizing and smoothing intestines, anticancer, anti-aging, and improving immunity [4]. In Southwest China and Tibet, RVT is known as “Xigui” or “Dujiao-Danggui” [5] and is often used as a substitute for RAS for strengthening the spleen, nourishing the stomach and blood, regulating menstruation, relieving pain, and treating indigestion [5,6]. Moreover, the morphological features of RVT are similar to those of RAS, and most of the time, mixed and sold in the market. RVT is used more than RAS in daily nourishing diets in southwestern China, which usually appears in vegetable markets as a raw material for soup [5], and its price is more expensive than RAS. Therefore, RVT and RAS must be compared to ensure the effectiveness and suitability of RVT for its application.

*Vicatia thibetica* de Boiss is cultivated in Dali, Yunnan, Aba Prefecture, Sichuan, Tibet, and other provinces of China [6]. So far, there are few research reports on the comprehensive evaluation of chemical components and pharmacological activities of RVT [2,5,6], which limit the in-depth development and utilization of RVT. Thus, to ensure the effectiveness of RVT and RAS, they must be tested and analyzed for chemical composition and activity. The present study was conducted to investigate and compare the chemical constituents (polysaccharides, flavonoids, total coumarin, ferulic acid, chlorogenic acid, polyphenols, protein, amino acids, and volatile components) of RVT and RAS. Moreover, the biological activities (antioxidant, scavenging of nitrite, inhibition of tyrosinase, inhibition of thrombin, and coagulation factor Xa) were also analyzed and compared. The present study provides a basis for the further comprehensive development and utilization of *Vicatia thibetica* de Boiss.

## 2. Results and Discussion

### 2.1. Chemical Composition of RVT and RAS

Angelica contains abundant active components, and an in-depth analysis of its phytochemical composition is needed to assure its quality. Therefore, this study systematically analyzed the polysaccharide (DPC), total coumarins (DTC), total phenol (DTP), total flavonoid (DTF), chlorogenic acid (CA), ferulic acid (FA), protein, and volatile oil (DVO) contents of RVT-1, RVT-2, and RAS (Table 1). These data showed that the chemical contents of RVT exhibited significant differences compared to RAS. For instance, the DPC content in RVT-1 (20.44%) and RVT-2 (21.83%) was significantly higher (*p* < 0.01) than those observed in RAS (19.53%). This could be one of the reasons for the sweet taste of RVT. The research results showed that the DTP and DTF contents found in RVT-1 and RVT-2 were remarkably higher than those in RAS. For instance, the DTP content in RVT-1 (8.59 mg/g) was about four times that in RAS (2.29 mg/g). Interestingly, the DTF content in RVT-1 (6.53 mg/g) was 13 times higher than that of RAS (only 0.54 mg/g). These data indicated that RVT contains more polysaccharides, phenols, flavonoids, and proteins than RAS. Flavonoids are a large class of active ingredients and have many important pharmacological activities such as antioxidant, antiviral, hepatoprotective, anticancer, anti-inflammatory, etc. [7]. Polysaccharides are one of the main biologically active components in Angelica [8,9]. Phenolic components in Angelica can reduce oxidative stress and prevent cancer [3,10]. So RVT has broad application prospects to be utilized in functional food and pharmaceutical industries.

Angelica plants generally contain coumarin compounds, which have anti-hypertensive, antiarrhythmic, antiasthmatic, antiosteoporosis, antibacterial, anti-inflammatory, anticancer, and anti-oxidative functions [3,11]. However, the DTC content in RVT-1 (5.98 mg/g) and RVT-2 (5.52 mg/g) were significantly lower (*p* < 0.01) than those recorded in RAS (6.29 mg/g). Both ferulic acid and chlorogenic acid are very important organic acids in Angelica. Among them, ferulic acid is also one of the quality control indicators of *Angelica sinensis* in the Chinese Pharmacopoeia [3,12]. Ferulic acid has antithrombotic, blood lipid-lowering, uterine contraction inhibition, and nitrite clearance activities [13,14]. As can be seen from Table 1, the content of ferulic acid in RVT-1 is 0.59 mg/g, and only 0.39 mg/g in RVT-2, which were significantly lower (*p* < 0.01) than RAS (1.20 mg/g). Chlorogenic acid is a depsidic acid and can be metabolized by hydrolysis and methylation to generate ferulic acid, which has antibacterial, antioxidant, and anti-inflammatory effects [15,16]. Chlorogenic acid was found in RVT-1 (0.46 mg/g) and RVT-2 (0.42 mg/g) and was remarkably lower than those in RAS (0.95 mg/g).

Protein and amino acids are the nutritional components of plant-based drugs. The protein content is shown in Table 1, and the types and contents of amino acids are shown in Table 2. These data showed that the content of both protein and amino acids contained in RVT-1 (19.59% and 23.56%) and RVT-2 (19.00% and 18.44%) were found to be higher than that in RAS (10.52% and 15.02%). A total of 17 types of amino acids were determined in RVT and RAS. Among these, the contents of 14 types of amino acids were higher in RVT-1 and RVT-2 than that in RAS. It is worth mentioning that the content of lysine in RVT-1 is extremely high, reaching 7.49%, which is found to be 6–7-fold higher than that in RAS. Lysine can regulate the metabolic balance of the human body and can improve the body’s absorption of calcium, accelerate bone growth, promote growth and development, increase appetite, reduce disease, and enhance physical fitness [17]. In addition, the content of aspartic acid and glutamic acid was higher in RVT-1 and RVT-2 than those in RAS, and both are umami amino acids, which can improve the taste [18,19]. This could be one of the reasons why people prefer to use RVT for soup. Tyrosine was not detected in RVT-1. These results indicate that RVT is a nutrient-rich material containing high levels of proteins and amino acids that are much higher than other edible plants [18].

The content of volatile oil is one of the quality control indicators of Angelica in the Chinese Pharmacopoeia [12]. The volatile oil has anti-inflammatory, antioxidant, analgesic, and other pharmacological activities [20,21]. Additionally, the type and content of volatile components are the main factors affecting the odor of plants. Therefore, in this study, the supercritical CO_2_ fluid extraction method was used to obtain volatile oil. The content of volatile oil is shown in Table 1. The content of volatile oil in RVT-1 (0.37%) and RVT-2 (0.33%) was much lower than those contained in RAS (1.53%). The present study also analyzed the types and contents of volatile components in RVT-1, RVT-2, and RAS, and the results are presented in Table 3. Thirty-four compounds were identified from the volatile oil of RAS. These include four aldehyde compounds, accounting for 7.75% of the total composition of volatile oil. In addition, 17 terpene compounds accounted for 18.31% of the total composition of volatile oil. The contents of both butyraldehyde and 4-Isopropylbenzaldehyde were higher. Among terpenes, the higher content was found for 1R-α-Pinene (1.11%), 3-ethyl-1,5,5-trimethyl cyclohexene (1.34%), D-limonene (5.27%), 3,7-dimethyl-1,3,7-octatriene (4.29%), β-Cedrene (1.13%). Six kinds of esters, including senkyunolide A (0.11%), 3-butylidenephthalide (9.79%), Z-ligustilide (26.92%), E-ligustilide (3.95%), 3-N-butylphthalide (14.68%), and diisobutyl phthalate (2.27%) were also recorded. The total content of these esters compounds accounted for a total volatile oil 57.61% of the composition, and three kinds of fatty acids account for 3.22% of the total composition of volatile oil, of which the linoleic acid content is 2.67%. In addition, the volatile oil also contains heptacosane (1.09%), 2-methoxy-4-vinyl phenol (1.94%), and p-isopropyl toluene (0.24%). However, only 25 compounds were identified from the volatile oil in RVT-1 and RVT-2, including ten terpenes, four esters, five organic acids, and six kinds of other organic compounds. Interestingly, 3-N-butylphthalide in RVT-1 (55.22%) and RVT-2 (56.96%) accounted for two-thirds of the total volatile oil content from RVT, respectively. In addition, the content of 3-butylidenephthalide was also higher.

As indicated from the results (Table 3), there are 14 kinds of similar components in RVT-1, RVT-2, and RAS. Among these, the content of 3-N-butylphthalide was higher than those of RAS. In contrast, the content of total ligustilide in RVT-1 and RVT-2 is very low, which is much lower than that of volatile oil in RAS. Ligustilide is one of the components that impart the aroma and bitterness of authentic angelica. Hence RVT is less bitter than RAS. Therefore, the lower content of ligustilide in RVT could be one of the reasons for no bitterness, and it has a better taste than RAS. The volatile oil of RAS contained 20 ingredients that are not present in RVT-1 and RVT-2, of which 10 are more than 1%, namely butyraldehyde (3.82%), 1R-α-Pinene (1.11%), 3-ethyl-1,5,5-trimethyl cyclohexene (1.34%), D-limonene (5.27%), 3,7-dimethyl-1,3,7-octatriene (4.29%), 2-methoxy-4-vinyl phenol (1.94%), β-cedrene (1.13%), heptacosane (1.09%), 4-isopropyl benzaldehyde (3.23%), and diisobutyl phthalate (2.27%). There are 11 compounds present in RVT-1 and RVT-2 but not in RAS. Among them, 2,6-dimethyl-2,4,6-octatriene (8.89%), β-caryophyllene (2.03%), and oleic acid (2.26%) content are higher, while others are lower. In addition, the volatile oil composition was slightly different in RVT-1 and RVT-2.

### 2.2. Antioxidant and Nitrite Scavenging Activities of RVT and RAS

**Figure 1:**
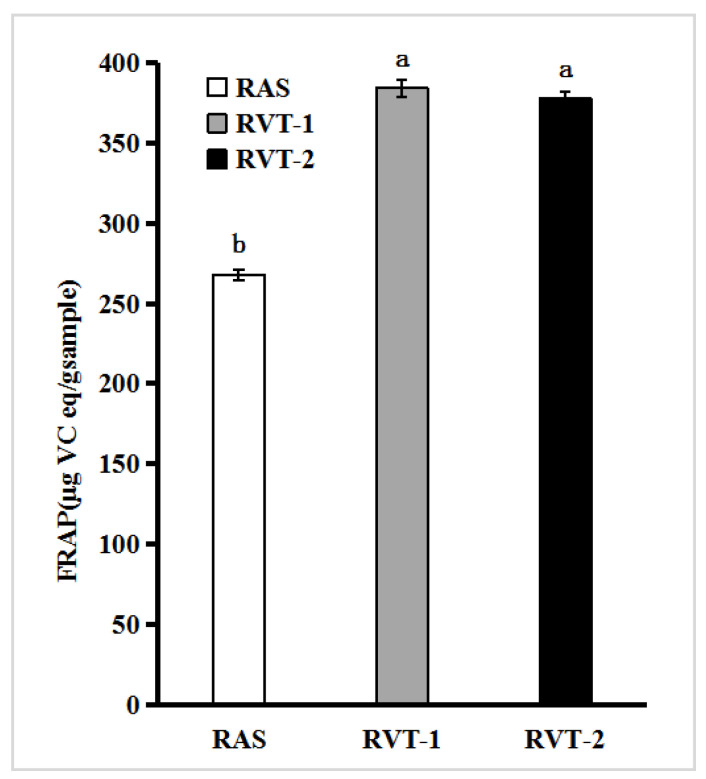
FRAP assays for RVT and RAS. Values with different letters within the same substance are significantly different (*p* < 0.05). All data were expressed as the means ± SD, *n* = 3.

A free radical can be defined as any molecular species capable of independent existence that contains an unpaired electron in an atomic orbital. Thus, these are unstable and highly reactive [22]. Under normal circumstances, free radicals are in a state of dynamic equilibrium, and the concentration is very low. However, when the balance is broken, in addition to causing aging, it will also cause a series of diseases, such as cancer, diabetes, cardiovascular disease, etc. [22]. The addition of antioxidants can effectively inhibit the peroxidative damage caused by excessive free radicals in the body, and some efficacy of *Angelica sinensis* is closely related to their ability to scavenge free radicals in the body. We carried out the ability of RVT-1, RVT-2, and RAS to scavenge DPPH free radicals, O_2_^−^ free radicals, and Ferric ion-reducing capacity (FRAP). The FRAP was used to evaluate the total antioxidant capacity of the samples. The reducing power of each sample is converted by the standard curve established by ascorbic acid(V_C_), and the total reducing capacity of each sample is represented by the equivalent content of V_C_. The results are depicted in Figure 1. The highest reducing power was recorded for RVT-1 (383.92 µg V_C_ eq/g_sample_), followed by RVT-2 (377.40 µg V_C_ eq/g_sample_), and the lowest total reducing power was recorded for RAS (267.69 µg V_C_ eq/g_sample_). It can be recorded that the total reducing power of the extracts of RVT and RAS was significantly different (*p* < 0.05). It can be seen that the potential reducing power was positively correlated with the content of the flavonoids and phenolics.

**Figure 2:**
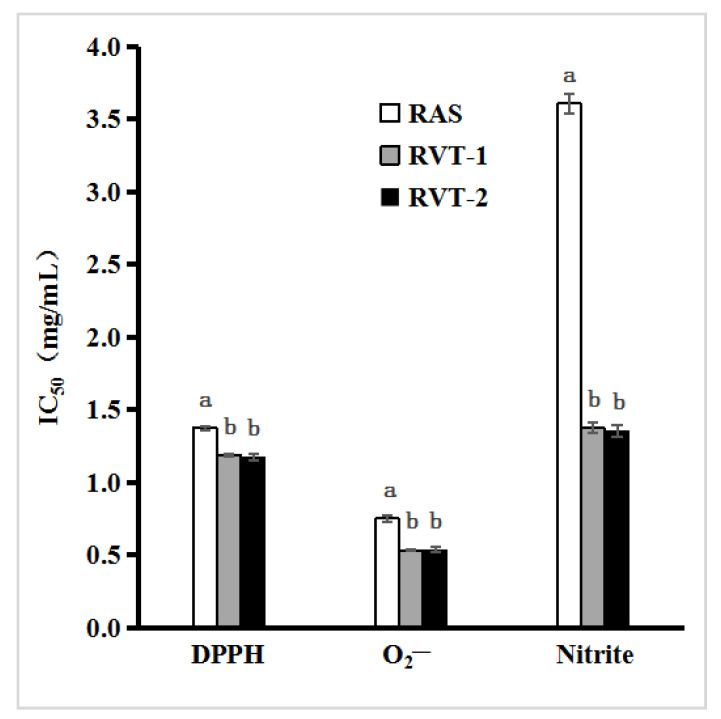
IC_50_ values of DPPH, O_2_^−^ radicals, and Nitrite inhibition for RVT and RAS. Values with different letters within the same substance are significantly different (*p* < 0.05). All data were expressed as the means ± SD, *n* = 3.

To evaluate the scavenging ability of the samples more accurately to DPPH and O_2_^−^ radicals, the IC_50_ values of DPPH and O_2_^−^ radicals are shown in Figure 2. The IC_50_ of the extracts of each sample for DPPH scavenging ability, the maximum was recorded for RAS (1.373 mg/mL). The IC_50_ of RVT-1 and RVT-2 are similar. The difference between the two is not significant. It can be concluded that RVT has a higher scavenging ability of DPPH free radicals than RAS. It can be seen from the IC_50_ values of O_2_^−^ radicals that the IC_50_ of RVT-1 (0.532 mg/mL) and RVT-2 (0.537 mg/mL) were lower than RAS (0.750 mg/mL). The difference in IC_50_ between RVT and RAS was significant (*p* < 0.05), indicating that RVT has a stronger ability to scavenge O_2_^−^ radicals.

Nitrite and nitrosamine are recognized toxic substances and have always been the focus of food quality, environment, and feed industries, and the accumulation of nitrite in the body can cause cancer [23,24]. Meanwhile, the nitrite scavenging activities of RVT and RAS were analyzed. The results are shown in Figure 2. The IC_50_ of the nitrite scavenging power of each sample extract is similar to that of DPPH and O_2_^−^ radicals, which was significantly lower in RVT-1 (1.371 mg/mL) and RVT-2 (1.353 mg/mL than RAS (3.607 mg/mL). The results indicated the nitrite scavenging ability of RVT-1 and RVT-2 were better than RAS.

The above results of total reducing capacity, DPPH, O_2_^−^ radicals, and nitrite scavenging power indicated that both RVT and RAS have antioxidant activity and reduce the accumulation of nitrite in the body. Furthermore, the antioxidant and nitrite scavenging activity of RVT was significantly higher than RAS. Researchers found that the antioxidant and nitrite scavenging capacity was influenced positively by the flavonoids and polyphenols content [23,25]. Natural plants, vegetables, and fruits are rich in a variety of antioxidant substances, such as phenols and flavonoids, which can improve the SOD (superoxide dismutase) and catalase activity in the body and inhibit the formation of nitrite and nitrosamines [26]. While the polysaccharides, phenols, and flavonoids content in RVT are higher, which could be an important reason for its higher antioxidant and nitrite scavenging activity. These also indicate that both RVT has a stronger anti-aging effect and could be comprehensively utilized in health-promoting effects, and RVT is more suitable as a high-efficiency and low-toxic natural antioxidant than traditional Chinese medicine *Angelica sinensis* (RAS).

### 2.3. Tyrosinase Inhibitory Activity of RVT and RAS

**Figure 3:**
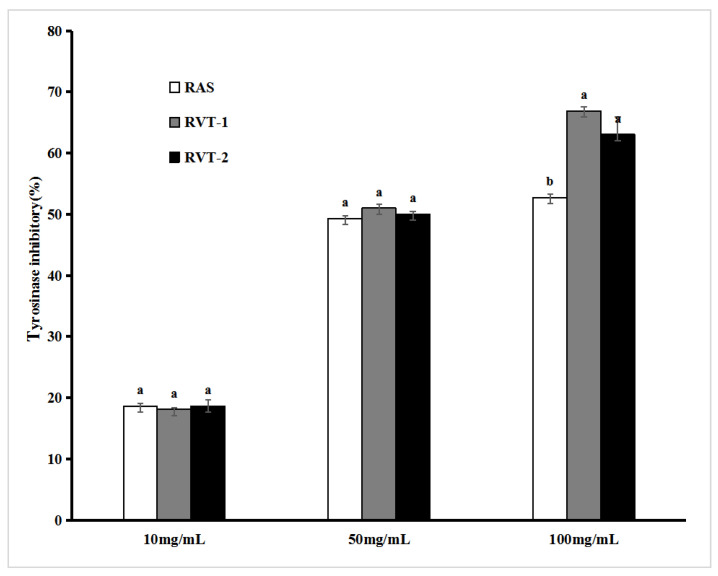
Tyrosinase inhibitory ability assays for RVT and RAS. Values with different letters within the same substance are significantly different (*p* < 0.05). All data were expressed as the means ± SD, *n* = 3.

Melanin is the main cause of human skin pigmentation [27]. The mechanism of action of most whitening raw materials is exerted by reducing the production of melanin, and inhibition of tyrosinase activity is one of the main strategies to reduce the production of melanin. There are many kinds of whitening skincare products using *Angelica sinensis* as active components in China. In this study, we measured the tyrosinase inhibitory ability of each sample extract to evaluate the whitening effect of RVT and RAS. Figure 3 shows the results of tyrosinase inhibitory activity at concentrations of 10 mg/mL, 50 mg/mL, and 100 mg/mL of the RVT-1, RVT-2, and RAS. The tyrosinase inhibitory ability gradually increased with an increase in the concentration. The tyrosinase inhibition activity of the RAS, RVT-1, and RVT-2 at concentrations of 10 mg/mL and 50 mg/mL was not statistically different, showing inhibition activity of 18.63%, 18.13%, and 18.63% at a concentration of 10 mg/mL for the RAS, RVT-1, and RVT-2, respectively, and at a concentration of 50 mg/mL showing 49.33%, 51.00%, and 50.03%, respectively. However, at a concentration of 100 mg/mL, a statistical difference (*p* < 0.05) was seen. The RVT-1 and RVT-2 have a stronger inhibition of tyrosinase activity by 66.90% and 63.03% than RAS (52.73%). Many studies have confirmed that polyphenols [28] and flavonoids [29] can inhibit tyrosinase activity, especially polysaccharides have a strong moisturizing effect [30]. RVT is rich in polysaccharides, flavonoids, and polyphenols, which could be a reason for RVT’s strong tyrosinase inhibitory activity. In addition, the tyrosinase inhibitory activity of RAS hardly increased beyond the concentration of 50 mg/mL, while RVT continued to increase until the concentration reached 100 mg/mL. Hence, the concentration of 100 mg/mL was considered the most effective among the tyrosinase inhibitory activity of RVT evaluated. The above results indicated that RVT has a strong whitening effect and quite good potential to be utilized in whitening skin care products.

### 2.4. In Vitro Thrombin and Factor Xa Inhibitory Activities of RVT and RAS

Thrombin and coagulation factor Xa (FXa) have unique and critical roles in the coagulation process and are key enzymes in blood coagulation and thrombosis [31]. Inhibition of FXa not only inhibits the occurrence of extrinsic coagulation but also blocks endogenous coagulation [32]. It can also exert an antithrombotic effect, ensuring normal hemostatic function. Thrombin is in the final stage of the coagulation cascade and can promote the conversion of fibrinogen to insoluble fibrin to play a hemostatic effect. The increase in thrombin plays an important role in thrombosis, and blocking their activity is the current treatment option. It is an important strategy for thrombotic diseases, and both have become drug targets for the treatment of thrombotic diseases [33]. As such, argatroban and rivaroxaban are widely used in clinical practice for the prevention and treatment of thrombosis [34]. Therefore, the inhibitory effect of the extract on the activity of thrombin and FXa can indicate the level of its promotion of blood circulation. In our study, we selected low-concentration (4 mg/mL) and higher-concentration (20 mg/mL) extracts to evaluate the ability of each sample to have blood-activating ability by inhibiting thrombin and FXa.

**Figure 4:**
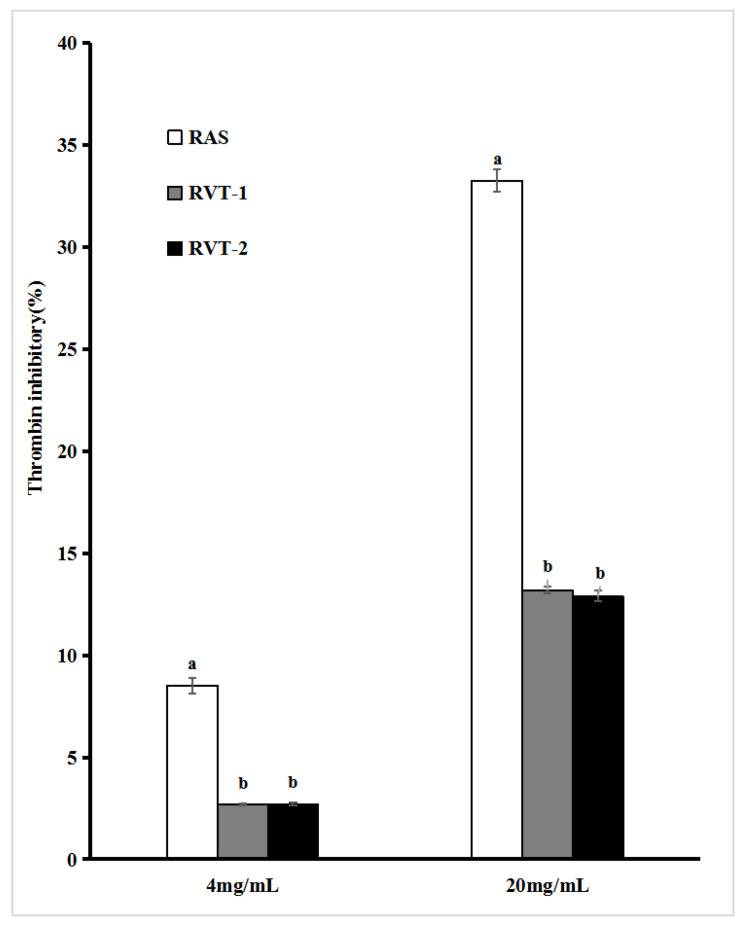
Thrombin inhibitory ability assays for RVT and RAS. Values with different letters within the same substance are significantly different (*p* < 0.05). All data were expressed as the means ± SD, *n* = 3.

The results of thrombin inhibitory activity are shown in Figure 4, and significant differences (*p* < 0.05) could be observed in RVT and RAS at concentrations of 4 mg/mL and 20 mg/mL. The RVT-1 and RVT-2 had a lower response with 2.68% and 2.71 % inhibitory activity at a concentration of 4 mg/mL, respectively, which of a quarter of RAS (8.51%), and they still showed lower activity (13.19% and 12.89%, respectively) at concentrations of 20 mg/mL, which was less than half of RAS (33.24%).

**Figure 5:**
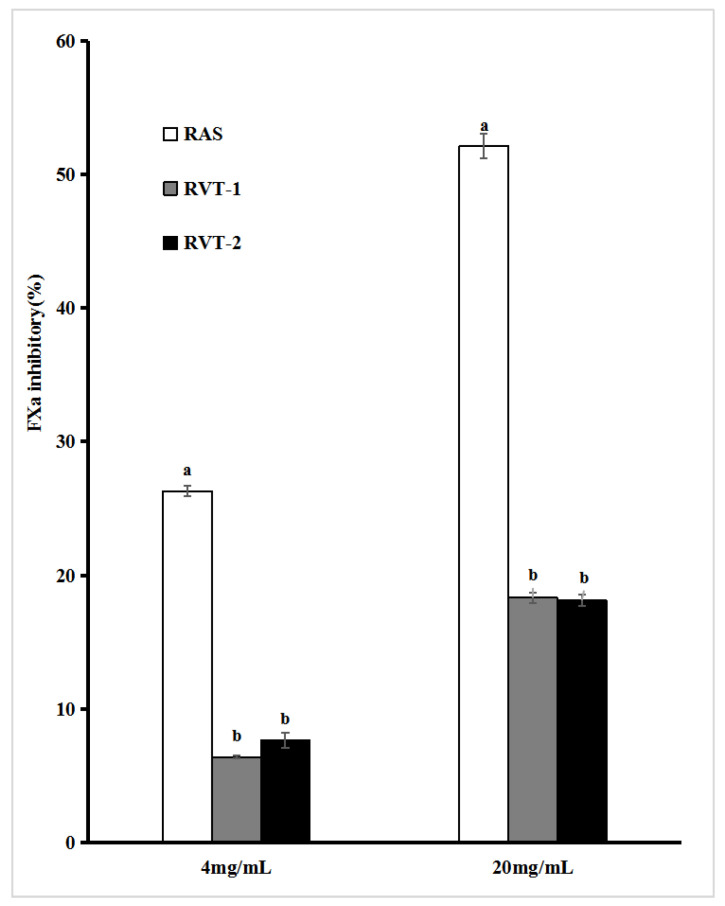
FXa inhibitory ability assays for RVT and RAS. Values with different letters within the same substance are significantly different (*p* < 0.05). All data were expressed as the means ± SD, *n* = 3.

**Figure 6:**
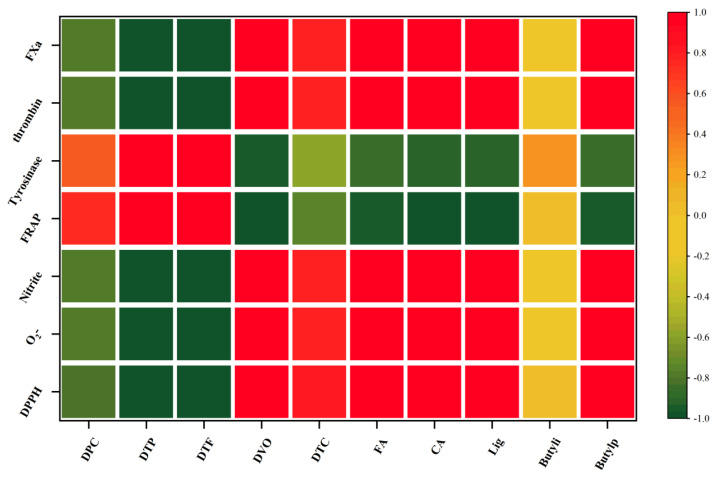
Heat map of correlation analysis of the chemical compounds and biological activity.

**Figure 7:**
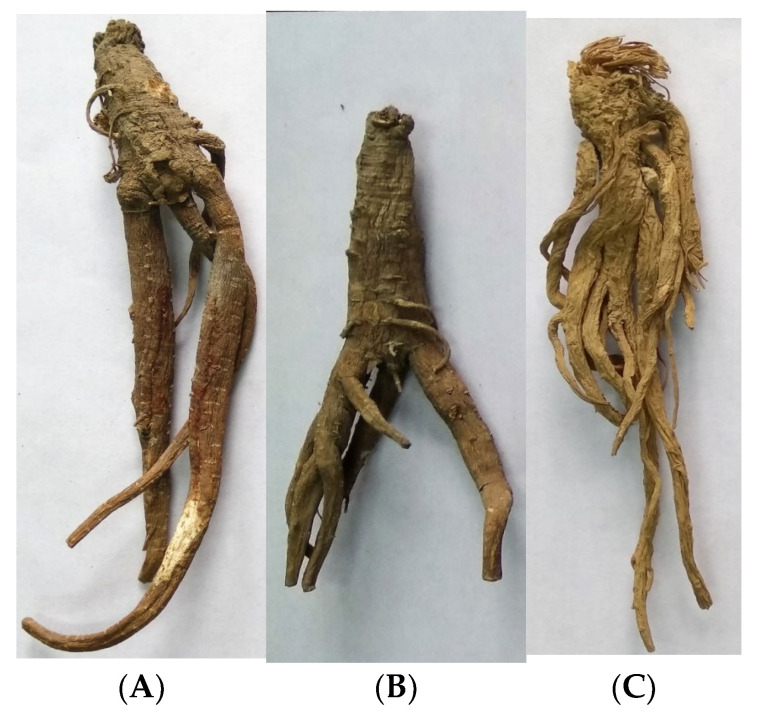
Morphological features of RVT-1 (**A**), RVT-2 (**B**), and RAS (**C**).

In terms of the inhibitory activity of each sample extract on FXa (Figure 5), at lower concentrations (4 mg/mL), RAS had a significantly higher (*p* < 0.05) FXa inhibition rate (26.30%) than RVT-1 (6.38%) and RVT-2 (7.65%). At higher concentrations (20 mg/mL), the FXa inhibition activity was observed in RAS, with a value of 52.13%, about three times higher than that observed from RVT-1 extracts (18.31%) and RVT-2 (18.13%). RVT has a lower FXa inhibitory activity, which could be related to the low content of ligustilide and coumarins in RVT. Some studies showed that ligustilide and coumarins exerted an anticoagulant effect [35,36]. In addition, it can be seen from Figure 6 and Figure 7 that compared with the thrombin inhibitory activities, the extracts of each sample can exert a “blood-activating” effect by inhibiting coagulation FXa to a greater extent. This was consistent with the findings of Lin Jia et al. [37].

### 2.5. Correlation Analysis

The correlation analysis was conducted to investigate the relationships between various main active ingredients and antioxidants, scavenging of nitrite, inhibition of tyrosinase, and blood-activating activities of RVT and RAS (Figure 6). As suggested by Pearson’s correlation coefficients of the IC_50_ value of scavenging DPPH, O_2_^−^ and nitrite, it was found that these are related to the total flavonoid (−0.974, −0.985, and −0.992, respectively, *p* < 0.01), total polyphenol (−0.972, −0.988 and −0.990, respectively, *p* < 0.01), and polysaccharide contents (−0.809, −0.768 and −0.789, respectively, *p* < 0.05). FRAP was also related to total polyphenols and total flavonoids (0.995 and 0.996, respectively, *p* < 0.01) and polysaccharides (0.752, *p* < 0.05). Thus, the higher amount of coumarins, total phenol, and flavonoids showed stronger antioxidant capacity [38]. According to the correlation analysis between tyrosinase inhibition rate and compounds, DTP and DTF were positively correlated with skin whitening (0.966, *p* < 0.01). This finding would greatly help health-promoting bioactivities and increase the phytochemical relevance for the better application of RVT. Thrombin and FXa inhibition assay are mainly related to DVO, CA, FA, ligustilide, and 3-N-butylphthalide content (*p* < 0.01), and ligustilide showed the highest correlation, and total coumarins (0.776, *p* < 0.05). The results suggested that volatile oil, chlorogenic acid, ferulic acid, and total coumarins were the main active components exerting blood-activating ability [13,21,35,37].

## 3. Materials and Methods

### 3.1. Materials

Radix *Vicatia thibetica* de Boiss was obtained from Luoza County, Tibet (RVT-1), and Heqing County, Yunnan Province (RVT-2), China. Radix *Angelica sinensis* (RAS) was obtained from Min County, Gansu Province, China. Each sample was dried at 50 °C, vacuumed, and stored in a sealed container until analysis. Samples were ground into powder and passed through a 50-mesh sieve before analysis. All samples were identified by Professor Chen Yuan of Gansu Agricultural University, China. The voucher specimens (GSNYSYHE-1019, GSNYSYHE-1020, GSNYSYHE-1021) are stored in the Herbarium of Chinese Herbs of Gansu Agricultural University, China. The morphological features of the different samples are shown in Figure 7.

### 3.2. Chemicals and Reagents

Standard chemicals were used, such as ferulic acid, chlorogenic acid, D-anhydroglucose, rutin, gallic acid, osthole, and other standard products with purity greater than 98%, purchased from China Foods and Drugs Institute for Testing, China. Ninhydrin and amino acids were purchased from Sigma Reagent Company, St. Louis, MO, USA. Other reagents were purchased from Shanghai Sinopharm Chemical Reagent Co., Ltd., Shanghai, China.

### 3.3. Phytochemical Analysis

The polysaccharide content was determined by the sulfuric acid-phenol method [39]. Briefly, the glucose standard curve was drawn with the mass concentration of D-anhydrous glucose as the abscissa (x) and the absorbance value as the ordinate (y). The linear regression equation of the standard curve was y = 0.0081x − 0.0039, R^2^ = 0.999. Referring to the extraction method established by Tian et al. [39], the sample treatment was slightly modified. Briefly, 1.0 g of each sample was accurately weighed, 100 mL of absolute ethanol was added and soaked overnight, filtered, and the filtrate was evaporated. The final volume of 50 mL was made with distilled water. The extract was shaken well and underwent ultrasonication (180 W, 40 kHz) for 40 min, reflux at 90 °C for 1 h, and centrifuge at 4000 r/min for 10 min. The supernatant was collected, and the absorbance was recorded at 490 nm after dilution.

The total coumarin content was determined by the spectrophotometry method, and the standard curve was drawn with osthole as the standard compound (y = 0.0549x − 0.0029; R^2^ = 0.9994). For each sample, 1.0 g of powder was accurately weighed, and 30 mL of anhydrous ethanol was added. The mixture was ultrasonicated (180 W, 40 kHz) for 30 min, heated and refluxed in a water bath for 30 min, and filtered. The absorbance was recorded at 320 nm after dilution.

The total polyphenols content was determined by the Folin phenol colorimetric method [40], and the standard curve was drawn with gallic acid as the reference standard (y = 3.7514x + 0.006, R^2^ = 0.9991). For each sample, 1.0 g of powder was accurately weighed, add 50 mL of 70% ethanol was added. The mixture was ultrasonicated (180 W, 40 kHz) for 30 min, heated to reflux in a water bath for 30 min, and filtered. The final volume was increased to 50 mL using 70% ethanol. The absorbance was recorded at 760 nm.

The total flavonoid content was determined by the aluminum chloride colorimetric method [40], and the standard curve was drawn with rutin as the reference substance (y = 10.39x + 0.0014, R^2^ = 0.9998). Briefly, the sample powder was accurately weighed (1.0 g), and 30 mL of 85% ethanol was added. The mixture was ultrasonicated (180 W, 40 kHz) for 30 min and heated under reflux in a water bath for 30 min. The filtered extract was made up to 50 mL with 85% ethanol. The absorbance was measured at a wavelength of 510 nm after dilution.

### 3.4. Determination of Ferulic Acid and Chlorogenic Acid

**Figure 8:**
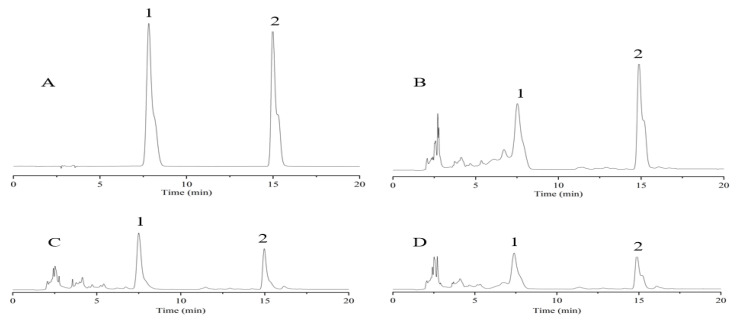
HPLC chromatograms of RVT and RAS. (**A**) Standards; (**B**) RAS; (**C**) RVT-1; (**D**) RVT-2; 1, chlorogenic acid; 2, ferulic acid.

The quantitative analysis of ferulic acid and chlorogenic acid was performed by an Agilent1260 HPLC (Agilent Technologies Inc., Santa Clara, CA, USA). Chromatographic separation was performed on a Kromasil-C_18_ column (250 mm × 4.6 mm, 5 µm). The mobile phase consists of 0.05% acetic acid and the gradient elution: 0–9 min, 10–17% acetonitrile; 9–17 min, 17% acetonitrile; 17–18 min, 17–10% acetonitrile. The flow rate was set at 1.0 mL/min, and the detection wavelength at 320 nm. The column temperature was set at 30 °C, and the injection volume was at 10 μL. The linear regression equation is expressed as a standard product (chlorogenic acid: y = 26.317x + 260.7, R^2^ = 0.9997; ferulic acid: y = 568.28x − 428.8, R^2^ = 0.9997). The HPLC chromatograms are shown in Figure 8.

The recovery rates of sample addition were 101.7% and 102.15%, respectively. The sample processing method refers to the 2020 edition of the Chinese Pharmacopoeia [12], with a slight modification. Each sample powder is 0.2 g, put into a conical flask, and 20 mL of 70% methanol was added precisely, extracted ultrasonically for 15 min, and heated to reflux for 30 min. The extract was kept on the stand, and the supernatant was filtered before analysis.

### 3.5. Determination of Proteins and Amino Acids

Protein content was determined using the Lowry method. The regression equation (y = 0.0065x + 0.14, R^2^ = 0.9993) was drawn with bovine serum albumin as the standard, and the absorbance of the sample was measured at a wavelength of 650 nm. The determination of amino acids was completed by SykamS433 amino acid analyzer (Germany), referring to GB 5009.124-2016 “Determination of Amino Acids in Food Safety National Standard” (China) [41].

### 3.6. Essential Oil Analysis

The extraction of volatile oil in the sample was carried out using HA121-50-05 supercritical extraction (Jiangsu Huaan Supercritical Extraction Co., Ltd., Jiangsu, China). Briefly, 200 g of sample powder was taken, the extraction temperature was set at 41 °C, the extraction pressure was at 30 MPa, and the extraction time was 2 h. The CO_2_ flow rate is set at 25 L/h, separation kettle I (pressure 8 MPa, temperature 50 °C), separation kettle II (pressure 6 MPa, temperature 35 °C), calculate the mass of the oily liquid obtained to calculate the volatile oil yield.

The components of volatile oil were separated by 6890GC-5973N MSD gas mass spectrometer (Agilent Technologies Inc., Santa Clara, CA, USA) using an OV1707 quartz capillary column (60 m × 0.25 mm, 0.5 μm). Gas chromatographic conditions are: the inlet temperature was set at 250 °C; no split; the carrier gas was He; the injection volume was set at 1.0 μL; the temperature program was adopted, and the initial temperature was set at 40 °C for 2 min, and the temperature was increased to 150 °C at a rate of 3 °C/min for 10 min retention, 4 °C/min heated to 220 °C for 8 min, 4 °C/min heated to 250 °C for 1 min). Mass spectrometry conditions are: the chromatography-mass spectrometry interface temperature is 280 °C, ion source temperature is 230 °C, electron energy is 70 eV, and scanning range is 30–500 m/z. The NIST03 standard spectral library was used for searching, and the relative content of each component was calculated using area normalization.

### 3.7. Antioxidant Activity

Antioxidant activity was assessed by mixing 3 g of each sample into a conical flask, and 100 mL of 70% methanol was added precisely. Subsequently, the conical flask was put into KQ2200DE ultrasonic device and extracted for 30 min (180 W, 40 kHz). The contents were boiled to reflux for 30 min, and the extract was transferred to a 100 mL volumetric flask and brought up to volume.

#### 3.7.1. FRAP Assay

Referring to the method of Wang et al. [38], 0.5 mL of each sample extraction 70% methanol solution after proper dilution was mixed with 5 mL of FRAP working solution (prepared for current use) in a water bath at 37 °C. After 30 min, the absorbance of the reaction solution was measured at 593 nm. The standard curve was drawn with different concentrations of ascorbic acid (V_C_) solutions (y = 0.0024x + 0.1269 R^2^ = 0.9992), and the total reducing power of each sample was represented by the equivalent content of V_C_ in a 1 g sample (μg V_C_ eq/g_sample_).

#### 3.7.2. DPPH Scavenging Assay

RVT and RAS extraction 70% methanol solutions were diluted into solutions of different concentrations, and 2 mL of each diluted solution was mixed with 2.0 mL of DPPH methanol solution. The absorbance was measured at 517 nm [42]. Antioxidant activity was expressed as IC_50_ of scavenging DPPH free radicals. According to the clearance rate of DPPH for each sample at different concentrations, IC_50_ was calculated using the probability regression analysis of SPSS.

#### 3.7.3. O_2_^−^ Scavenging Assay

This analysis was based on the pyrogallol auto-oxidation method [42]; 4 mL of sample extraction 70% methanol solutions with different concentrations were added to 5 mL of Tris-HCl buffer (0.05 M, pH = 8.2) that had been preheated in a water bath at 25 °C for 20 min. Then 1 mL of 3 mM pyrogallol solution (preheated in a 25 °C water bath for 20 min) was added, shaken, mixed quickly, and placed in a 25 °C water bath for 5 min. Immediately 1 mL of 10 M HCl solution was added to stop the reaction, and the absorbance was measured at 322 nm. The absorbance was measured at the same volume, and the sample was replaced by the same volume of distilled water as a blank control. Antioxidant activity was expressed as IC_50_.

### 3.8. Nitrite Scavenging Activity

Under weakly acidic conditions, nitrite was diazotized with p-amino benzene sulfonic acid and then coupled with naphthalene ethylenediamine hydrochloride to synthesize a purple-red dye. The absorbance was measured at 538 nm. Referring to the method of Jun Liu et al. [23]. with the following modifications, To mix 3.0 g of RAS and RVT sample powder with 50 mL of 70% methanol. The mixture was ultrasonicated for 30 min and then heated under reflux for 30 min. A total of 1 mL of sample extraction 70% methanol solution with different concentrations was mixed with 1 mL of 1 mM NaNO_2_ solution, 8 mL of sodium citrate buffer solution (pH = 3.0), and 2.0 mL 0.4% p-amino benzene sulfonic acid was added. The mixture was left to stand for 5 min, then 1.0 mL 0.2% naphthalene ethylenediamine hydrochloride was added and left to stand for 15 min. The absorbance was measured at 540 nm, the sample was replaced with the same volume of distilled water as blank control, and the nitrite-scavenging ability was expressed as IC_50_.

### 3.9. Inhibition of Tyrosinase Activity

The determination of tyrosinase inhibitory activity followed the method of Adrielly Á. Menezesde Sá et al. [43], with a slight modification. Briefly, each sample powder (10.0 g) was mixed with 70% methanol (250 mL) and extracted ultrasonically (180 W, 40 kHz) for 30 min, then heated under reflux for 1 h. The sample solution was concentrated to 0.1 g/mL by a rotary evaporator. A volume of 100 µL sample extraction 70% methanol solutions of different concentrations, 70 µL of L-tyrosine solution, and 70 µL of phosphate buffer (20 mM, pH 6.8) were added to the test tube, and mixed well, incubated in a water bath at 30 °C for 10 min, and then 30 µL of tyrosine was added to the test tube. The enzyme solution (500 U/mL, Sigma-Aldrich Trading Co., Ltd, Shanghai, China) was added to the mixed solution and incubated in a water bath at 30 °C for 30 min, and the absorbance was measured at 490 nm. The phosphate buffer solution was used instead of the sample as blank control, and kojic acid was used as a positive control. The tyrosinase inhibition rate was calculated as follows:

Inhibition rate (%) = 100 × [(A_blank_ − A_sample_)/A_blank_]

### 3.10. Thrombin and Factor Xa Inhibitory Activities

Referring to the methods of Jablonka et al. [44] and Wang Ying et al. [45], with minor modifications, mix 2.0 g of RAS and RVT sample powder with 50 mL of 70% methanol. The mixture was ultrasonicated for 30 min and then boiled for 30 min. A volume of 30 µL of bovine thrombin solution (1 mg/mL, Sigma-Aldrich Trading Co., Ltd, Shanghai, China) or coagulation factor Xa (0.5 U/mL, Beijing Aidehawk Co., Beijing, China) was added to 150 µL of 70% methanol-extracted samples (concentration: 4 mg/mL and 20 mg/mL). The mixture was incubated at 25 °C for 10 min, then 75 µL of thrombin chromogenic substrate S-2238 (concentration: 10 mg/mL) or S-2765 (concentration of 5 mg/mL) was added. After mixing, the mixture was incubated at 25 °C for 30 min, and a 20% formic acid solution was added to stop the reaction and measure the absorbance at 405 nm. The enzyme solution was replaced by 0.9% NaCl aqueous solution as blank control. The inhibition rates of coagulation FXa are calculated as the above equation.

### 3.11. Data Analysis

The SPSS software V. 19.0 was used for all statistical analyses. Significant differences between mean values were determined using one-way ANOVA at different significance levels, and Duncan’s test was used to identify significant differences. In addition, Pearson’s correlation was performed using SPSS software, and then a heat map drawing was performed with Origin 2019b. All experiments were conducted in triplicate, and IC_50_ values were determined by probit regression analysis.

## 4. Conclusions

In this study, we analyzed and compared the chemical composition and antioxidant, scavenging nitrite, promoting blood circulation, and inhibiting tyrosinase activities of roots from *Vicatia thibetica* de Boiss and *Angelica sinensis*. The *Vicatia thibetica* de Boiss root was rich in polysaccharides, polyphenols, flavonoids, coumarins, proteins, and amino acids. Especially polyphenols, flavonoids, proteins, glutamic acid, and lysine contents are very high. Meanwhile, the antioxidant, scavenging nitrite, and inhibiting tyrosinase activities are stronger than in traditional Chinese medicine Angelica. Thus, *Vicatia thibetica* de Boiss, as a unique national medicine, has great potential for drug development. Our present work will provide evidence of the application value of arguable *Angelica sinensis* alternatives (*Vicatia thibetica* de Boiss) by exploring its chemical composition and biological activity. Further, the applications of *Vicatia thibetica* de Boiss as functional foods, cosmetics, and medicine are recommended. Further, research needs to be carried out for comprehensive development and utilization of *Vicatia thibetica* de Boiss and its clinical and safety aspects.

## Figures and Tables

**Table 1:** Polysaccharide (DPC), total Coumarins (DTC), total phenol (DTP), total flavonoid (DTF), chlorogenic acid (CA), ferulic acid (FA), protein and volatile oil (DVO) contents.

Samples	DPC (%)	DTC (mg OS/g_sample_)	DTP (mg GA/g_sample_)	DTF (mg RU/g_sample_)	CA (mg/g)	FA (mg/g)	Protein (%)	DVO (%)
RVT-1	20.44 ± 0.45 ^bB^	5.98 ± 0.03 ^bB^	8.59 ± 0.02 ^aA^	6.53 ± 0.05 ^aA^	0.46 ± 0.01 ^bB^	0.59 ± 0.01 ^bB^	19.59 ± 0.22 ^aA^	0.37 ± 0.03 ^bB^
RVT-2	21.83 ± 0.36 ^aA^	5.52 ± 0.13 ^cC^	7.80 ± 0.15 ^bB^	5.77 ± 0.15 ^bB^	0.42 ± 0.01 ^cC^	0.39 ± 0.02 ^cC^	19.00 ± 0.43 ^aA^	0.33 ± 0.04 ^bB^
RAS	19.53 ± 0.16 ^cC^	6.29 ± 0.07 ^aA^	2.29 ± 0.19 ^cC^	0.54 ± 0.01 ^cC^	0.95 ± 0.01 ^aA^	1.20 ± 0.01 ^aA^	10.52 ± 0.21 ^bB^	1.53 ± 0.05 ^aA^

Abbreviations: OS, osthole; GA, gallic acid; RU, rutin. These data are presented as the means ± SD. RVT-1, *Root of Vicatia thibetica* de Boiss from Luoza County, Tibet; RVT-2, *Root of Vicatia thibetica* de Boiss from Heqing County, Yunnan Province; RAS, *Root of Angelica sinensis* (Oliv.) Diels. Within each column, the different superscripted small and capital letters indicate significant and highly significant differences at *p* < 0.05 and *p* < 0.01, respectively. All data were expressed as the means ± SD, *n* = 3.

**Table 2:** Amino acid contents of RVT and RAS.

Amino Acid	Amino Acid Content (%)		
	RVT-1	RVT-2	RAS
Asp	1.96	2.13	1.15
Thr	0.63	0.66	0.54
Ser	0.74	0.72	0.43
Glu	3.10	3.12	2.06
Gly	0.76	0.79	0.64
Ala	0.76	0.78	0.67
Cys	0.06	0.04	0.04
Val	0.97	1.01	0.67
Met	0.29	0.29	0.15
Ile	0.72	0.70	0.55
Leu	1.35	1.34	0.92
Tyr	—	0.38	0.33
Phe	1.24	1.25	0.47
His	0.45	0.50	0.27
Lys	7.49	1.14	1.02
Arg	2.62	3.16	4.89
Pro	0.41	0.43	0.23
Total content	23.56	18.44	15.02

**Table 3:** Volatility chemical composition of RVT and RAS.

No	Compounds	MF	MW	RAS		RVT-1		RVT-2	
				RT (min)	A%	RT (min)	A%	RT (min)	A%
1	Butyraldehyde	C_4_H_8_O	72.11	5.454	3.82	—	—	5.460	2.01
2	Acetal	C_6_H_14_O_2_	118.17	5.868	0.13	—	—	5.874	3.51
3	1R-α-Pinene	C_10_H_16_	136.23	8.108	1.11	—	—	—	—
4	α-Pinene	C_10_H_16_	136.23	8.279	0.72	—	—	—	—
5	2,6-dimethyl-2,4,6-Octatriene	C_10_H_16_	136.23	—	—	9.527	0.16	9.522	8.89
6	β-Pinene	C_10_H_16_	136.23	11.792	0.27	—	—	—	—
7	β-Caryophyllene	C_15_H_24_	204.35	—	—	11.920	2.03	12.009	0.11
8	β-Myrcene	C_10_H_16_	136.23	12.127	0.15			—	—
9	(+)-Ledene	C_15_H_24_	204.35	—	—	16.407	0.31	—	—
10	β-selinene	C_15_H_24_	204.35	—	—	17.388	0.24	17.376	0.09
11	α-Farnesene	C_15_H_24_	204.35	—	—	19.775	0.14	—	—
12	3-ethyl-1,5,5-trimethyl cyclohexene	C_10_H_16_	136.23	21.382	1.34	—	—	—	—
12	6-n-butyl-1,4-cycloheptadiene	C_11_H_18_	204.35	—	—	21.394	0.61	21.394	0.19
13	Amyl ketone	C_11_H_22_O	170.29	—	—	21.875	0.43	—	—
14	D-limonene	C_10_H_16_	136.23	24.378	5.27	—	—	—	—
15	p-isopropyltoluene	C_10_H_14_	136.23	27.331	0.24	—	—	27.339	4.65
16	g-Terpinene	C_10_H_16_	136.23	28.141	0.11	—	—	—	—
17	3,7-dimethyl-1,3,6-Octatriene	C_10_H_16_	136.23	28.476	0.26	—	—	28.482	0.25
18	3,7-dimethyl-1,3,7-Octatriene	C_10_H_16_	136.23	28.939	4.29	—	—	—	—
19	1,4-cyclohexadiene-1,2-dicarboxylic anhydride	C_8_H_6_O_3_	150.13	35.728	0.14	—	—	35.758	1.03
20	4-ethyl-methyl ester Benzoic acid	C_9_H_10_O_2_	150.17	39.436	0.07	—	—	—	—
21	2,6-dimethyl-2,4,6-Octatriene	C_10_H_16_	136.23	46.073	0.42	—	—	—	—
22	Guaiene	C_15_H_24_	204.35	50.061	0.51	—	—	—	—
23	α-caryophyllene	C_15_H_24_	204.35	—	—	—	—	51.559	1.31
24	2-Methoxy-4-vinylphenol	C_9_H_10_O_2_	150.17	52.667	1.94	—	—	52.685	0.72
25	β-Farnesene	C_15_H_24_	204.35	53.300	0.45	—	—	—	—
26	2,4,5-trimethyl-Benzaldehyde	C_10_H_12_O	148.20	53.818	0.57	—	—	—	—
27	β-Chamigrene	C_15_H_24_	204.35	54.366	0.19	—	—	—	—
28	β-Bisabolene	C_15_H_24_	204.35	55.115	0.66	55.036	0.18	—	—
29	(+)-β-Cedrene	C_15_H_24_	204.35	56.826	1.13	—	—	—	—
30	(+)-α-Longipinene	C_15_H_24_	204.35	57.301	0.57	—	—	—	—
31	γ-Elemene	C_15_H_24_	204.35	59.133	0.86	—	—	59.127	0.86
32	Senkyunolide A	C_12_H_16_O_2_	192.25	62.720	0.11	—	—	—	—
33	Heptacosane	C_27_H_56_	380.73	64.839	1.09	—	—	—	—
34	4-Isopropylbenzaldehyde	C_10_H_12_O	148.20	69.369	3.23	—	—	—	—
36	3-Butylidenephthalide	C_12_H_12_O_2_	188.22	70.818	9.79	70.819	14.19	70.813	5.63
37	Z-Ligustilide	C_12_H_14_O_2_	190.24	73.047	26.92	73.071	2.76	73.607	0.79
38	E-Ligustilide	C_12_H_14_O_2_	190.24	73.260	3.95	73.259	0.15	—	—
39	3-N-Butylphthalide	C_12_H_14_O_2_	190.24	75.435	14.68	75.437	55.22	75.421	56.96
40	Diisobutyl phthalate	C_16_H_22_O_4_	278.38	75.707	2.27	—	—	—	—
41	Pentadecanoic acid	C_15_H_30_O_2_	242.40	—	—	71.451	0.41	—	—
42	Margaric acid	C_17_H_34_O_2_	270.45	—	—	76.204	0.29	76.189	0.23
43	n-Hexadecanoic acid	C_16_H_32_O_2_	256.42	77.571	0.48	77.576	4.21	77.556	1.63
44	oleic acid	C_18_H_34_O_2_	282.46	—	—	79.061	2.26	79.032	1.65
45	Linoleic acid	C_18_H_32_O_2_	280.45	79.884	2.67	79.835	8.13	79.984	3.31

Abbreviations: MF, Molecular formula; MW, Molecular weight; RT, Retention times; A%, Peak areas; ND, not detected.

## Data Availability

Not applicable.

## Sample Availability

Samples of the compounds are not available from the authors.
